# Mechanisms of Geomagnetic Field Influence on Gene Expression Using Influenza as a Model System: Basics of Physical Epidemiology

**DOI:** 10.3390/ijerph7030938

**Published:** 2010-03-10

**Authors:** Valeriy Zaporozhan, Andriy Ponomarenko

**Affiliations:** Odessa State Medical University, Valekhovsky lane 2, 65082, Odessa, Ukraine; E-Mail: rector@odmu.edu.ua

**Keywords:** Solar cycles, electromagnetic fields, pair radical reactions, Cryptochrome, gene expression, NF-kB, influenza, epidemiology, circadian transcription

## Abstract

Recent studies demonstrate distinct changes in gene expression in cells exposed to a weak magnetic field (MF). Mechanisms of this phenomenon are not understood yet. We propose that proteins of the Cryptochrome family (CRY) are “epigenetic sensors” of the MF fluctuations, *i.e.*, magnetic field-sensitive part of the epigenetic controlling mechanism. It was shown that CRY represses activity of the major circadian transcriptional complex CLOCK/BMAL1. At the same time, function of CRY, is apparently highly responsive to weak MF because of radical pairs that periodically arise in the functionally active site of CRY and mediate the radical pair mechanism of magnetoreception. It is known that the circadian complex influences function of every organ and tissue, including modulation of both NF-κB- and glucocorticoids- dependent signaling pathways. Thus, MFs and solar cycles-dependent geomagnetic field fluctuations are capable of altering expression of genes related to function of NF-κB, hormones and other biological regulators. Notably, NF-κB, along with its significant role in immune response, also participates in differential regulation of influenza virus RNA synthesis. Presented data suggests that in the case of global application (example—geomagnetic field), MF-mediated regulation may have epidemiological and other consequences.

## 1. Introduction

Influenza pandemics have been threatening to people for many centuries. Each influenza pandemics bring on huge financial and people losses. Timely and correct epidemiological prognosis could attenuate the damage. But it is not an easy task, because numerous factors influence the occurrence of influenza (like any other) epidemics, among them:

- virus evolution dynamics;
- host’s sensitivity;
- conditions for viral transmission, *etc.*

Therefore, it is always difficult (if possible at all) to make an exact epidemiological forecast. However, reliability of any prognosis increases with an increase in our understanding of underlying mechanisms and influencing factors. There is an opinion that solar activity can impact epidemic processes. Chronology of influenza pandemics in 20th century (1946–1947, 1957 and 1968) suggested existence of 11-years periodicity in flu pandemics. At the same time, cycles of solar activity usually have 11-years periodicity as well. Could it be just a simple coincidence? In the beginning of 20th century, Russian scientist A. Chizhevsky suggested correlation of some biological processes on the Earth with cycles of solar activity [1]. But possible mechanisms of such interrelation are still not completely understood.

In this paper, we support an idea of a definite role of electromagnetic and Geomagnetic fields in biological regulation, including regulation of gene expression patterns in any living being that can directly influence flu epidemic process and any other biological phenomena. Existence of “electromagnetic bio-regulation” would easily explain solar influences on the Biosphere. We’re going to expound our views and pertinent data in the literature in the following order:

- To bring attention to periodicity as a common feature of numerous biological processes and to discuss the nature of corresponding regulatory influences;
- To show theoretical possibility of bio-regulatory effects of magnetic fields;
- To outline some signaling pathways capable of implementing bio-regulatory (including genome-regulatory) functions of electromagnetic fields;
- To summarize our knowledge about Geomagnetic field, its principle parameters and sources of variation;
- To review possible evidences of regulatory influence of Solar cycles and corresponding Geomagnetic field perturbations on flu epidemic process;
- To describe probable mechanisms of Solar cycles and Geomagnetic field regulatory influences on virus-host interactions and other biological processes.

## 2. Periodicity as a Common Feature of Numerous Biological Processes and Nature of Corresponding Regulatory Influences

Life entails a spectrum of natural rhythms. Periodicity is a typical feature of numerous biological processes and life itself on various levels of its organization: subcellular and cellular (segmentation clock, cell cycle, transcription factors’ activity oscillations, *etc.*) [2–4], tissue and organ (oscillations in electrical activity of the brain, heartbeats), organism (circadian rhythms) [5,6], in populations of organisms and in Biosphere (seasonal changes, *etc.*). Some authors have collected evidence of “Solar-dependent” rhythm in biological processes [7–10]. In 1936, A. Chizhevsky concluded: “Life is a phenomenon… It lives due to dynamics; each oscillation of organic pulsation is coordinated with the cosmic heart in a grandiose Whole of nebulas, stars, the sun and the planet.” [1].

There is evidence that some epidemic processes have rhythmic nature as well [11,12]. It is possible to notice periodicity in influenza epidemic occurrence. For example, E.D. Kilbourne reported a 10–11 year periodicity in the occurrence of influenza pandemics and proposed that this is due to a periodicity in antigenic shifts [13]. R.E. Hope-Simpson noted coincidences between flu pandemics and maxima of solar activity, which occur with an approximately 11-year period [14]. These observations were continued, developed and statistically tested later on [15–17]. Do there exist any universal mechanisms able to drive and regulate this biological and Biosphere periodicity in its numerous manifestations? To answer this question, let us recognize some characteristic elements of biological periodic processes in general.

Machinery of many periodic processes includes several typical components, among them:

- pacemaker;
- regulatory signals emitted by pacemaker;
- receptors of the regulatory signals in the controlled constituent.

A review of modern biomedical knowledge shows that regulatory signals in many known biological processes are of an electromagnetic nature: heartbeat, breathing, peristaltic motions of guts – all these manifestations of life exist due to transmittance of electrical and chemical signals in corresponding neuronal chains. Among less evident regulatory functions of the electromagnetic fields (**EMF**), there are the direction of cell differentiation [18], regulation of the orientation and frequency of cell division and wound healing rate, control of nerve growth and cell migration [19], among others. The BioInitiative Report [20] contains a reasonable statement: “Human beings are bioelectrical systems. Our hearts and brains are regulated by internal bioelectrical signals”. At the same time, our environment is permeated by EMFs of natural and artificial origin. The main sources of the natural EMFs are atmospheric electricity, the Earth’s magnetic field and its occasional perturbations caused by interaction between the geomagnetic field and solar wind plasma. Environmental exposures to EMFs can interact with fundamental biological processes and there may be no lower limit at which EMF exposures affect us [21].

Such an important and universal role of electromagnetic interactions in biology is not unexpected since there are four known types of interactions in nature of which only two can exercise effects over a distance that exceeds the dimension of an atomic nucleus. Mentioned “long-distance” interactions are Gravity and Electromagnetic **(EM)** interactions, and there are no others. It is the basic reason explaining why this particular EM force puts in motion and governs biological processes, implementing signaling and regulatory functions in every living creature and in the whole Biosphere. This concept has been convincingly substantiated in the current literature [19,22–25].

**Figure 1.**
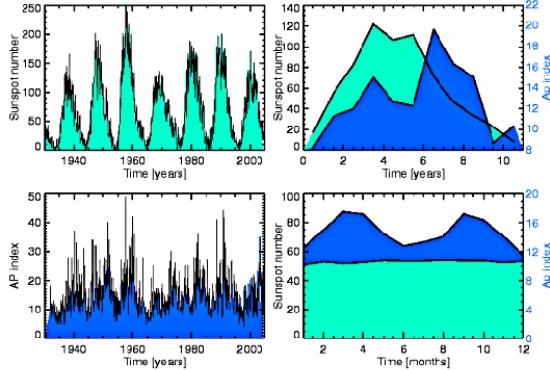
Periodicities in solar and geomagnetic activity. The left panels show time series of the monthly values of the sunspot number and the geomagnetic A_p_ index. The top right panel shows the solar cycle variation present both in the geomagnetic and solar records, showing peak geomagnetic activity during the declining phase of the solar cycle. The bottom right panel shows the semiannual variation in the geomagnetic data not visible in the solar records. (From [26], reproduced by the kind permission of the author and the publisher. The source database: [27]).

It is important to note that the main part of the known solar regulatory signals influencing Biosphere are of **electromagnetic nature**:

- in most evident cases it is solar light, which represent electromagnetic waves of definite wavelength, and has numerous biological effects, including regulation of circadian rhythms in living beings.
- in the case of cycles of Solar activity it is alterations of the Earth’s Magnetic field, caused by fluctuations in levels of solar ionizing radiations and solar energy output (Figure 1).
- it is also worthy to mention a slowly-varying Microwave emission from the solar corona.

## 3. Theoretical Possibility and Evidences of Bio-Regulatory Effects of Weak Magnetic Fields

**Figure 2.**
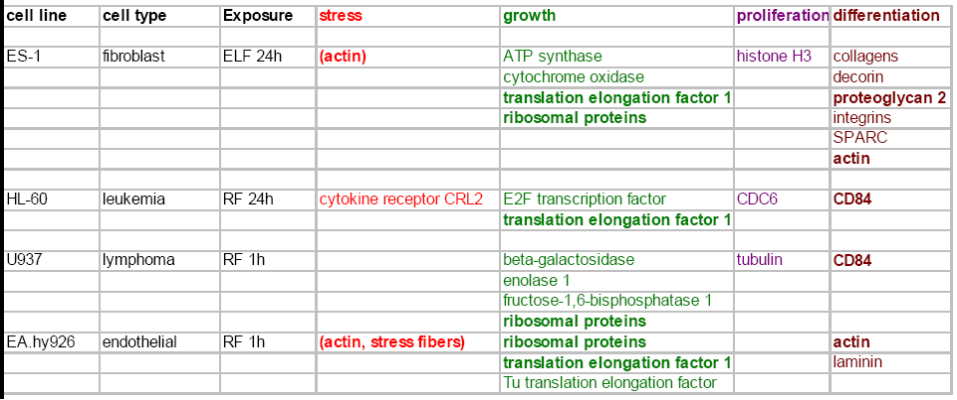
Summary of genes regulated as a consequence of cell exposure to radio-frequency (RF) and extremely low frequency (ELF) electromagnetic field*. (From [29], reproduced by the kind permission of Dr. Maercker). * *Presented results derived from comparative gene expression studies and are not proven in alternative assays yet.*

Recent genetic studies of different cell lines and primary cells provide convincing evidence of the bio-regulatory capabilities of magnetic and electromagnetic fields. Distinct changes in gene expression have been detected in cells after exposure to either radio-frequency EMF, extremely low frequency (ELF) and static fields [28,29] (Figure 2). Understanding of mechanisms of these genetic effects attracts much scientific interest. Most intriguing are mechanisms of the bioregulatory effects of weak magnetic and electromagnetic fields. The *information* conveyed by electromagnetic radiation (rather than heat) appears to cause these biological changes [20]. Thus, the most important and certainly the most universal are non-thermal interactions of electromagnetic fields with cellular systems. Experimental and epidemiological data as well as theoretical considerations suggest that predominantly magnetic, not the electric constituent of the EMFs, is mainly responsible for biological effects of the EMFs, by virtue of its greater ability to penetrate biological tissues [30,31]. Besides, in contrast to EMF, magnetic fields are not attenuated by most common materials. Therefore, we will focus on possible mechanisms of non-thermal bioregulatory effects of weak magnetic fields.

In spite of the large number of works that cover this issue, the primary biological “receiver” of weak electromagnetic radiation, as well as the complete sequence of events—the cause-effect relationships between the physical signal and biochemical or physiological responses—are still controversial questions. However, convincing hypotheses and explanations of pertinent mechanisms have been presented in [19,28,32–34], among others. Theoretically, both electrical and magnetic signals can cause non-thermal biological effects, genuinely:

- electrical fields are able to interact with charged molecules, surfaces and electric dipoles of biomolecules, and
- magnetic fields can interact with magnetic dipoles of electron spins, whose carriers are paramagnetic molecules, metal ions and ion-radicals [35].

Electrons within atoms and biomolecules clearly play an essential role in mechanisms of bioregulatory effects of weak magnetic fields because electrons directly participate in biochemical reactions as well as being sensitive to magnetic fields because of their magnetic dipole moment [36].

The majority of mechanisms proposed thus far to explain bioregulatory function of extremely weak magnetic fields can be assigned to one of three groups:

1. Mechanisms following the “plasma membrane hypothesis”, which proposes that the cell membrane is a primary biological receiver of magnetic signals, in that it responds to magnetic field influences by changes of its potential, and modulates the distribution and activity of integral membrane proteins and ion channels (e.g., Ca^2+^ channels). However, the primary molecular interaction remains unclear.
2. Free radical mechanisms. The basis of these mechanisms is the phenomenon that magnetic fields can increase the lifetime of free radicals, *i.e.*, stabilize them for longer. This results in an increase in free radical concentration in cell compartments, and hence biological changes including activation of signaling cascades (reviewed in [33]).
3. The ion resonance model, which implicates the combined action of the ELF MF and a weak static magnetic field (for example - geomagnetic field) [37,38].

In describing possible roles of the electron paramagnetic resonance in mechanisms of biological responses to EMF exposure, Fursa has concluded that “Electrons and nuclei, which possess magnetic moment, are the “magnetic antennae” in any biosystem, including human beings. They are able to receive and radiate electromagnetic energy selectively depending of the field’s frequency (ν) and gyromagnetic ratio (γ)” [34].

In our view, most genetic and other bioregulatory effects of weak magnetic fields (including geomagnetic field) can be achieved *via* the pair-radical mechanism of biological magnetoreception, first proposed by Schulten and coauthors [39]. Therefore, our main goals are to analyze the pair-radical mechanism, to propose and substantiate an expanded version of signaling pathways it can operate, and to outline corresponding medical-biological implications.

### 3.1. Radical Pairs and Pair Radical Reactions

Radicals are very reactive paramagnetic chemical species because they have an odd number of electrons and consequently unpaired electron spins that may be found in one of two spin states, designated ↑ or ↓. To create radicals usually requires the input of external energy. Electron excitation by light is the main route to create radicals. When they are formed from diamagnetic precursors, radicals are created in pairs. A pair of radicals generated from the same diamagnetic precursor is called a geminate radical pair (G-pair).

**Figure 3.**
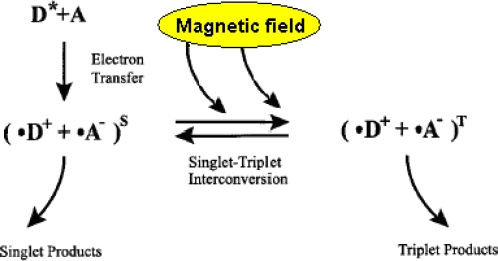
Reaction scheme for a radical pair reaction with magnetic field-dependent reaction products. The radical pair is generated by an electron transfer from a donor molecule D to an acceptor molecule A. An external magnetic field affects interconversion between singlet and triplet states of the radical pair. In these conditions, an applied weak magnetic field will result in an increased transient conversion of the radical pair into the triplet state, causing triplet products to be formed more rapidly and in higher yield (**S**—singlet state of the radical pair, **T**—triplet state of the pair). (Reproduced from [32] by permission of the publisher, with minor modification).

The radical pair is a short-lived reaction intermediate. Two radicals formed in tandem may have their unpaired electron spins either antiparallel (↑↓, a singlet state—**S**, with zero total electron spin) or parallel (↑↑, a triplet state—**T**, with unit spin). The radical pairs have unique properties. Since each electron spin has an associated magnetic moment, the interconversion and chemical fates of the S and T states can be influenced by internal and external magnetic fields (Figure 3). The minimum requirement for a radical pair reaction to be sensitive to an external magnetic field is that at least one of the S and T states undergoes a reaction that is not open to the other, usually as a consequence of the requirement to conserve spin angular momentum [40].

### 3.2. Key Features of a Radical Pair Magnetoreceptor

As mentioned above, radical pair reactions were first proposed as a magnetoreceptor by Schulten and coauthors. The unique property of the radical pairs is that their chemical fate is largely controlled by weak (in the microTesla range) magnetic fields *via* their spin correlation. It was shown that the magnetic field effects on chemical reactions are much stronger in viscous solution, as in micelles.

Magnetic field effects in reactions of spin-correlated pairs are related to singlet-triplet transitions in these pairs, which can switch between the singlet and triplet channels of the reaction. These transitions usually take the form of dynamic oscillations (beats) between the singlet and triplet states of a pair, with the frequencies depending on both the strength of external magnetic field (as a consequence of Zeeman effect) and specific parameters of the radicals, such as g-factors and hyperfine coupling constants [41]. Through their effects on the evolution of the overall spin in a radical pair, hyperfine and Zeeman interactions (and consequently external magnetic field strength) may control the reaction yields into different reaction channels. The dependence of a reaction yield on the external magnetic field strength is called a MARY spectrum (**MARY—**Magnetically Affected Reaction Yields) [42]. Several models describing magnetic field influence on kinetics of enzymatic reactions that involve free radical-dependent chemistry have been elaborated. These models demonstrate that even subtle alterations in radical pair recombination kinetics induced by weak magnetic fields might lead to measurable effects on enzyme activity [43,44]. Study of magnetic field effects on radical pair reactions that may have biological consequences is an important task [45].

Thus, the radical pair mechanism is a plausible way in which weak magnetic field variations can affect chemical reactivity, allowing radical pairs containing substances that can function as chemical/biological magnetic sensors.

## 4. Signaling Pathways Capable of Implementing Bio-Regulatory (Including Genome-Regulatory) Functions of Magnetic Fields

To prove the existence of the effects of magnetic fields on genome regulation, it is necessary to identify immediate receptors of the magnetic regulatory signals and their role in the genetic machinery. We propose a theory that magnetic fields induce definite genetic effects due to the existence of **magnetic field-sensitive transcription factor repressors** capable of regulating biological activity of organisms through epigenetic mechanisms. These substances are proteins of the Cryptochrome/Photolyase family. Valuable information regarding magneto-sensitivity of Cryptochromes (**CRY**) and their biological responses to weak magnetic fields was obtained with the plant model, *Arabidopsis thaliana* [46]. If plants—for which magnetic responses have no apparent function—are sensitive to external magnetic fields, it is conceivable that other CRY-containing species are also sensitive [47].

### 4.1. Cryptochromes: Ancient Regulatory Proteins Sensitive to Electromagnetic Radiation and Magnetic Fields

Cryptochromes are blue-light photoreceptor flavoproteins (50–70 kDa) found in plants, bacteria, insects, and animals [48]. They contain two non-covalently bound chromophores, a redoxactive flavin adenine dinucleotide (**FAD**), and a light-harvesting cofactor.

**Figure 4.**
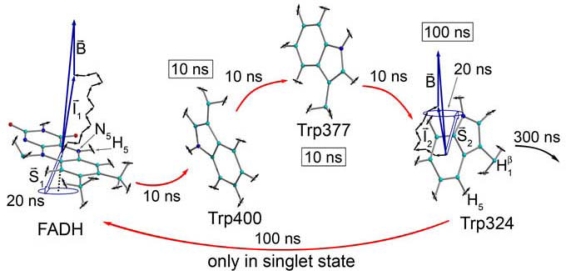
Scheme of the magnetic field effect on the radical pairs between FADH and tryptophan in cryptochrome active site. The unpaired electron spins (S_1_ and S_2_) precess about a local magnetic field produced by the addition of the external magnetic field B with contributions I_1_ and I_2_ from the nuclear spins on the two radicals. The spin precession continuously alters the relative spin orientation, causing the singlet (anti-parallel) to triplet (parallel) interconversion, which underlies the magnetic field effect. Electron back-transfer from a tryptophan to FADH quenches cryptochrome’s active state. However, this back-transfer can only take place when the electron spins are in the singlet state, and this spin-dependence allows the external magnetic field, B, to affect cryptochrome activation. *This image was made with VMD and is owned by the Theoretical and Computational Biophysics Group, an NIH Resource for Macromolecular Modeling and Bioinformatics, at the Beckman Institute, University of Illinois at Urbana-Champaign.* (From [51], reproduced by the kind permission of the authors and the publisher (Elsevier)).

Identification of a new cryptochrome class (cryptochrome DASH) in bacteria and plants suggests that cryptochromes evolved before the divergence of eukaryotes and prokaryotes [49]. It was proposed in 2000 that CRY could host magnetically sensitive radical pairs [32]. The process of cryptochrome signaling presumably involves conformational changes in the protein that promote interaction with its downstream signaling partners [48,50]. The most recent scheme concerning the magnetic field effects on CRY activity comes from [51], the concept is shown and described in Figure 4. It should be noted that the FADH-access cavity of the helical domain is predicted to be a functionally active site of the CRY molecule.

What, however, is known about localization and function of CRY proteins in the eukaryotic cell and in the whole organism? Cryptochromes are ubiquitously expressed in the organs and tissues of all organisms, and have a predominantly nuclear localization [48]. What is especially intriguing is that CRY protein function is related to cell genome expression regulation; they belong to the transcriptional repressors group [52,53]. Among known functions of CRY are the regulation of growth and development (in plants) and entrainment of circadian clocks [40]. They act as integral parts of the central circadian oscillator in animal brains and play an important role in mechanisms of circadian variations in expression of genes [54,55]. The circadian control system increases fitness and allows organisms to adapt to their physical and ecological environment. Therefore, animals with mutated a CRY gene have severe molecular and behavioral problems [56].

In plants, CRY proteins act as receptors controlling photomorphogenesis in response to blue or ultraviolet (UV-A) light. CRY are probably the evolutionary descendants of DNA photolyases, which are light-activated DNA-repair enzymes [48]. This suggests adoption of a transcription regulatory function (cryptochrome) by a redox repair enzyme (photolyase) during evolution. Presumably, CRY activity does not necessarily require light exposure.

With regard to the localization of CRY in selected organs, it is important to note the presence of CRY proteins in the supraoptic nucleus of the hypothalamus, which is the central regulator of periodicity of daily (circadian) rhythms. This means that cells of the supraoptic nucleus can emit and spread signals regulating functional activity of organs, tissues and whole organism. CRY proteins were also found in endocrine glands, fibroblasts and other cell types.

### 4.2. Cryptochrome-Mediated Pathways and Biological Effects

The above data clearly show the possibility that magnetic (including geomagnetic) fields can alter the functional activity of CRY proteins. To appreciate the possible effects of such alterations, we need to understand CRY-dependent signaling pathways.

**Figure 5.**
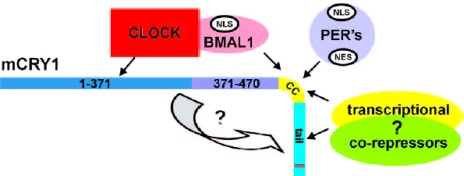
Representation of the co-operation of CRY molecule with the major cell cycle regulator-transcription factor heterodimer, CLOCK/BMAL1, transcriptional corepressors and PER proteins. (From [53], reproduced with permission from American Society for Microbiology).

Some mechanisms of genome-regulatory effects of CRY proteins have recently been described. The schematic image of co-operation of CRY with the major cell regulatory proteins is presented in Figure 5. In *Drosophila*, CRY interacts with key circadian clock proteins, period and timeless (PER and TIM), in a light-dependent manner [56]. In mammals, CRY inhibits the activity of the **CLOCK/BMAL1** heterodimer, which controls expression of hundreds of genes, including circadian clock regulators—genes ***Per*** and ***Cry*** [52,53]. It is believed that CLOCK/BMAL1-dependent rhythmic expression of numerous genes provides the explanation for circadian control of multiple physiological outputs [57]. Physical interaction between CRY and the PER-TIM protein complex is required for heat-mediated behavioural responses in *Drosophila* [56]. Taken together, these data indicates that CRY participates in regulation of various adaptive responses including responses to temperature and light variations.

**Figure 6.**
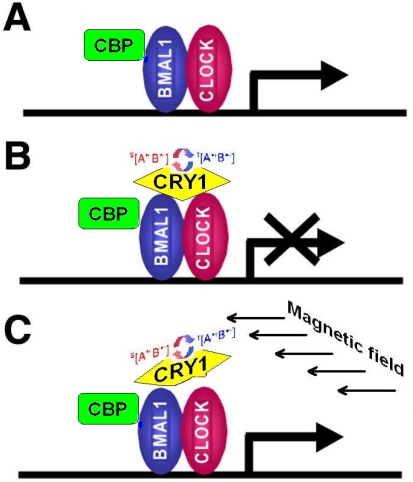
Scheme representing possible mechanisms of CRY bio-regulating activity. **(a)** CLOCK/BMAL1-heterodimer function as transcriptional activator. **(b)** interaction between CRY1 and CLOCK/BMAL1 causes CLOCK/BMAL1 to adopt a transcriptional repression function. **(c)** modification of CRY activity by weak external magnetic field and subsequent restoration of CLOCK/BMAL1 transcriptional activation function. **CBP**: CREB-binding protein.

According to [58], CRY1 and CRY2 bind directly to BMAL1 close to its C-terminus, which has been described as a potential interaction site for an important transcriptional coactivator, the CREB-binding protein (**CBP**), which has intrinsic histone acetyltransferase activity. Some authors suggest that CRY controls circadian gene expression by periodic disruption of CLOCK/BMAL1 interaction with histone acetyl-transferase (**HAT**), which results in alteration of HAT recruitment to specific promoter regions and correspondingly the repression of circadian gene expression [59]. Later, it was shown that CRYs themselves do not act as transcriptional inhibitors, but execute their repressor function by converting the CLOCK/BMAL1 complex from a transcriptional activator to a transcriptional repressor [60]. We have further developed this concept to propose mediatory functions of the CRY as primary “epigenetic sensors” of magnetic field fluctuations. Our conception of the CRY function as magnetic field-sensitive part of the epigenetic regulatory mechanism is shown in Figure 6. As already mentioned, external magnetic fields with specific parameters may modify the functional activity of CRY. In such cases, we can expect either abolishment of the CRY repressor function and restoration of CLOCK/BMAL1 transcriptional activation function (Figure 6(c)), or an increase of CRY transcription repressor activity (not shown).

This proposed mechanism perfectly explains several previously inexplicable data, such as alteration of circadian rhythms in laboratory animals and humans during extremely low frequency EM radiation exposure [61,62], and the pronounced increase in transcription in cells exposed to ELF EMF [63]. The latter authors also noticed changes in transcriptional and translational patterns induced by the ELF EM fields in exposed cells. It is important that field strength and frequency- and time-dependent “windows” relative to quantitative changes in specific transcripts were observed [64,65]. Taken together, these features confirm our suggestions concerning the circadian complex-related, pair-radicals-mediated mechanism of biological magnetoreception.

Thus, magnetic fields are able to influence the machinery related to circadian control of gene expression by means of CRY activity modulation. Moreover, it was shown that the CLOCK/BMAL1/CRY1-complex can block promoter activation by other non-circadian transcription factors, working as active transcriptional repressors [60]. The authors showed that daily variations in the sensitivity of normal cells and tissues to genotoxic stress induced by anticancer therapy correlate with CLOCK/BMAL1/CRY1 transcriptional activity. We propose that magnetic fields can implement this mechanism of active transcriptional repression *via* the CRY activity modulation, thereby influencing transcription of numerous genes. Such a concept is appropriate in explaining some previous findings, for example, the marked decrease in expression of mRNA for ALF1 and histone H3.3A in hippocampal neurons cultured under sustained exposure to static magnetic fields [66]. Significance of ascertaining the pertinent mechanisms was emphasized because “it appears that static magnetism may modulate cellular integrity and functionality through expression of a variety of responsive genes required for gene transcription and translation, proliferation, differentiation, maturation, survival, and so on” [66].

To summarize, CRY are unique bio-compounds, which combines sensory and bioregulatory functions. They act as mediators between living beings and their physical environment, providing a mechanism for both the reception of electromagnetic signals and the triggering (or entraining) of biological responses.

### 4.3. Ca^2+^-Operated Pathways and Ion Resonance Effects

Ca^2+^-mediated signaling represents another feasible target for regulatory influences of magnetic fields on biological processes. Much experimental data and corresponding theoretical considerations have accumulated on this issue (reviewed in [67]). We will briefly mention some results corroborating the main idea that external magnetic fields can modify some biological parameters and signaling pathways, including those of immunological and epidemiological significance.

**Figure 7.**
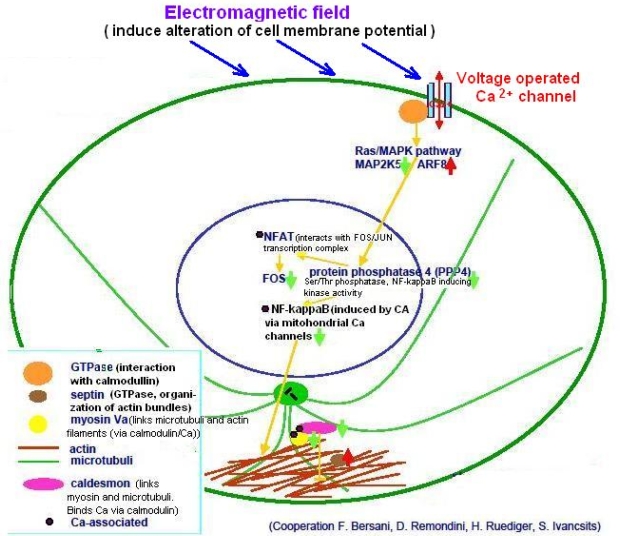
Possible mechanisms of the extremely low frequency EMF effects on transcription through Ca^2+^-operated pathways. (From [77], with modifications; reproduced with the kind permission of Dr. Maercker).

Calcium is the most universal signal used by living organisms to convey information for many different cellular processes. There are several well-known and recently identified proteins that sense and decode the calcium signal, and which are key elements in the nucleus in the regulation of the activity of various transcriptional networks (reviewed in [68,69]). Ca^2+^ has a central role in transcriptional responses [70]. Several Ca^2+^-dependent transcriptional pathways acting on cytokine genes have been described [71]. The d scheme presented in Figure 7) summarizes some effects of electromagnetic field on the Ca^2+^-dependent transcriptional network.

In spite of the many hypotheses and the theoretical considerations and experimental data that have accumulated in this field, the exact mechanisms of Ca^2+^-mediated biological effects of weak magnetic fields is not completely understood. Among feasible initial events of magnetic field-induced alterations in Ca^2+^-operated pathways there is modulation of the cell membrane potential and consequent changes in voltage-activated ion channel function. For example, Nuccitelli *et al.* [72] noted alterations in plasma membrane potential in tumor cells under the influence of magnetic fields of different types (static or pulsating) and intensities. The authors suggested that plasma membrane hyperpolarization may be part of the signal transduction chain determining the anti-apoptotic effect of magnetic fields. A four-fold increase in transmembrane calcium influx induced by ELF EMF was observed [73].

Specific frequencies of EMF have a differential effect on calcium ion activity. For example, it was demonstrated that radio frequency EM radiation exposure causes changes in calcium ion activity when the EM radiation is amplitude modulated, and only within a certain frequency of amplitude modulation: effects occurred for modulations of 6, 9, 11, 16 and 20 Hz, but no effects occurred for modulations of 0.5, 3, 25 and 35 Hz [74]. It was subsequently shown that both the intensity and the orientation of the earth’s magnetic field during exposure can alter these effects at specific frequencies. These and similar [75] results led to the development of ion resonance models for biological effects of EMF [37,38,74,76].

It was proposed that membrane-mediated Ca^2+^ signaling processes are involved in the mediation of the electromagnetic field effects on the immune system [78]. Application of low frequency electromagnetic fields produce parallel shifts in Ca^2+^ uptake and DNA replication intensity in stimulated lymphocytes [79]. Exposure of immune cells to static magnetic field resulted in decrease of phagocytic activity, inhibition of mitogenic response to Con A in lymphocytes and enhancement of apoptosis in thymic cells [80]. Together, the data demonstrate the possibility of Ca^2+^ signaling-mediated immunomodulating effects of exposure to magnetic fields.

Thus, calcium signaling and Ca^2+^-dependent transcriptional network represents another possible target of electromagnetic field influence on biological functions and host-pathogen interactions.

## 5. Brief Summary of Our Knowledge about Geomagnetic Field, its Principle Parameters and Sources of Variation

Earth currents flowing considerably deeper than at the Earth’s crust generate the Earth’s main magnetic field. Land geomagnetic force varies from 35 μT on the equator to 65 μT in regions close to Earth poles. Regular changes of solar activity and corresponding outer space plasma flow alterations induce global geophysical field fluctuations with a frequency range from 0,001 Hz to 10 Hz.

The various manifestations of solar activity are driven by the changing amount and distribution of magnetic flux in the Sun. Solar activity dynamics was first discovered through observations of sunspots. Reliable counts of sunspots, integrated into an index called International sunspot number or Wolf number, date back from the present to at least the beginning of the 18th Century. In 1946, a better index of solar magnetic activity was discovered: the 10.7cm Solar Flux. This index represents measurement of radio emission from plasma concentrations formed in the solar corona by magnetic fields [81,82].

**Figure 8.**
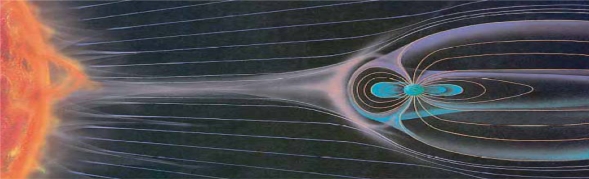
Schematic picture of “Sun wind” consisting of plasma fluxes erupted from the Sun’s surface and their interaction with Earth’s magnetic field.

Potent blows of solar plasma (solar eruptions) reaching the Earth cause geomagnetic storms. A mediatory function in this process plays Ionosphere – upper part of Earth’s atmosphere at altitudes ≈ 70–1,000 kilometers. Blows of high-energy cosmic radiation comprising electrons, protons, ultraviolet and gamma-rays, *etc.*, cause increase of ionization and intense currents in ionosphere (Figure 8). This generates potent electromagnetic fields throughout atmosphere, in oceans and even in the Earth crust. These fields and currents are able to influence any Earth organisms and their living conditions.

Duration of geomagnetic storm average between one or two days, at this period amplitude of magnetic field fluctuations may vary from 200–300 nT and until 1000 nT, which exceeds normal background values in different frequency bands by 5,000–10,000-times [83].

Intensity of industrial magnetic fields can amount up to 120 μT, but contribution of the industrial (artificial) magnetic fields in total average intensity of environmental magnetic field is rather small because of mutual compensation (cancellation) of industrial magnetic fields [84,85].

## 6. Possible Evidence of Regulatory Influence of Solar Cycles and Corresponding Geomagnetic Field Perturbations on Flu Epidemic Process

Now let’s return to the influenza epidemic process. Founder of HelioBiology, A. Chizhevsky, performed comparative retrospective analysis of flu epidemics with respect to solar activity cycles and came to following conclusions [1]:

- Occurrence of major influenza epidemics shows definite periodicity with an average period of 11, 3 years, equal to the period of Solar activity fluctuations;
- As a rule, significant influenza epidemics do not occur in years of minimum solar activity;
- Most major influenza epidemics occurred in time interval starting 2–3 years before and ending 2–3 years after solar activity maxima.

Several other authors also noticed correlation between cycles of solar activity and major influenza and other epidemics on the Earth [14–17,86]. Solar activity was estimated using reliable records of sunspot number dating back to at least 1700 and Solar Flux measurements started later on. Data about influenza pandemics in human history are available in several reviews: [87–89], and others.

**Figure 9.**
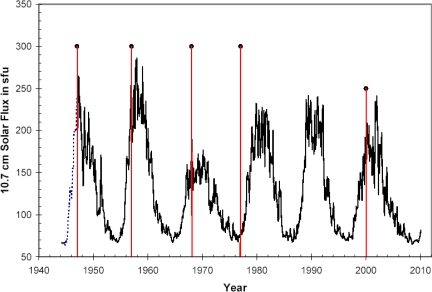
The 1946, 1957, 1968 and 1977 pandemics (shown as spikes) on a plot of the 10.7 cm Solar Flux index. The flux values prior to 1947 (shown dotted) are estimated from sunspot data. (From [86], reproduced by the kind permission of Dr. Tapping).

**Figure 10.**
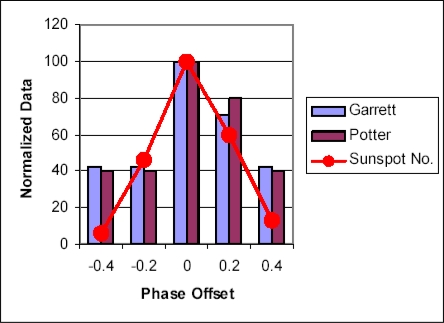
Distribution of Influenza Pandemics (according to different authors) as a function of phase offset from solar activity maximum. Data are normalized for a maximum of 100. The solar activity minimum lies at a phase offset of ±0.5. The activity maximum is at a phase offset of 0. The data from Garrett (1994) are shown in blue; those from Potter (1998) in violet. Sunspot Number is shown in red. (From [86], reproduced by the kind permission of Dr. Tapping).

Good visual and statistical materials concerning possible correlation between level of solar activity and influenza pandemics are contained in the work Tapping *et al.* [86]. The authors demonstrated that influenza pandemics often develop in periods of solar activity maximum (Figure 9). But there are some exceptions, such as the pandemic of 1977, which occurred close to activity minimum. Figure 10 presents a summarized correlation picture for all studied period (about 300 years), showing existence of rather strong interrelation between solar activity and activity of influenza epidemic process.

For the sake of objectivity, it should be noted that many authors negate existence of predictable pattern of pandemic periodicity [90]. But the main reason of the “unpredictable periodicity pattern” can be polygenic nature of the epidemic process, *i.e.,* existence of complicated interplay of numerous epidemic-predisposing and epidemic-preventing factors, including those of anthropogenic nature (migration, vaccination, *etc.*). These factors may mask regulatory influence of the solar activity cycles.

Solar cycles and geomagnetic field’s influences on human beings manifests not only through periodicity in global influenza infection spread. There are numerous observations supporting influence of the geomagnetic field perturbations on people’s health. Statistical analysis of hospital records showed that magnetic storms correlated with a significant rise of mental, nervous disorders and acute cardiovascular events (stroke and myocardial infarction) in urban populations [91,92]. Many authors noticed correlation between dynamics of immunological parameters in healthy people and Solar cycles: in years of maximum solar activity average leukocyte count decreased 1.5–1.67-times that in low solar activity phase and blood formula shifted towards lymphocytosis [93–95]. According to S. Tromp, solar activity may regulate human immunity; many years experience in donor blood examination allowed the researcher to conclude that the blood sedimentation rate varies with the sunspot cycle. Since this rate parallels the amount of albumin and gamma globulin, resistance to infection may also correlate with solar cycles [7].

All together, accumulated data support the idea about cycles of solar activity as the **pacemaker** of numerous biological phenomena including epidemics of some infectious diseases and dynamic changes in immunological parameters of living beings. As stated by Tapping and coauthors, “solar connection with pandemics may seem implausible at first sight; however solar modulation of many environmental parameters is now well established, and it is timely to revisit the issue of the connection between the occurrence of pandemics and the rhythm of solar activity” [86].

Exact mechanisms of such Solar regulating influences are not completely understood yet. In our opinion, infra-annual immune-regulatory and epidemic-predisposing solar effects to a great extent are mediated and can be explained by the concomitant geomagnetic field fluctuations. Indeed, bio-regulatory capabilities of the magnetic fields and pertinent mechanisms have been described in the first four paragraphs of this paper. Moreover, regular semiannual variations in the geomagnetic field (Figure 1, bottom right panel) greatly resemble seasonal variations in viral respiratory diseases sickness rate, which allows speculation about the possible role of the geomagnetic field fluctuations as both flu epidemics and seasonal flu- predisposing factors.

## 7. Probable Mechanisms of the Geomagnetic Field and Solar Cycles Influences on Biological Functions and Virus-Host Interactions

Following from the previous discussions, it is possible to predict some magnetic field-sensitive processes in living systems related to virus replication and host antiviral resistance. We can not consider all the pertinent mechanisms, but will focus here mainly on NF-κB-dependent pathways that play a key role in immune reactions, including response to infection.

NF-κB is a protein complex that acts as a transcription factor. It is widely expressed and positively regulates the expression of genes involved in immune responses, inflammation, proliferation, apoptosis and other cellular activities. Initial steps of these pathways include nuclear translocation of NF-κB dimers, where they bind to specific κB sequences in the promoter or enhancer regions of multiple target genes, including those encoding proinflammatory cytokines, adhesion molecules, interferon, pro-apoptotic molecules, *etc.* (reviewed in [96,97]).

Role of the NF-κB pathway in influenza infection is especially significant because NF-κB signaling plays an important role in differential regulation of influenza virus RNA synthesis [98]. These authors showned that overexpression of the p65 molecule (member of NF-κB subfamily) could activate influenza virus RNA transcription from the cRNA. And *vice versa*: siRNA-mediated knockdown of p65 protein significantly reduced influenza A virus replication and synthesis of most vRNA segments. NF-κB inhibitors:

- decrease influenza virus production in infected cells and viral gene expression;
- block early stages of the influenza virus life cycle;
- specifically decrease the vRNA level during influenza virus infection [98].

Is it possible that Solar cycles and geomagnetic field perturbations can influence the NF-κB signaling pathway or mechanisms of virus replication, or both? The answer is “Yes”. It was already shown that magnetic field can affect translation processes in bacteria [99]. Expression of virus DNA integrated into cells can be affected by EM fields as well. For example, exposure of SV40-transformed human fibroblasts to magnetic field resulted in increased levels of virally derived mRNA and virus-specific protein [100]. Understanding of mechanisms underlying these phenomena would be very important.

**Figure 11.**
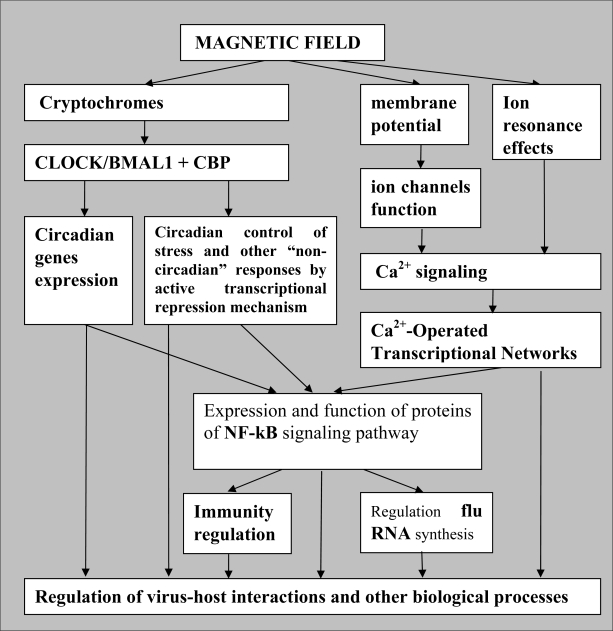
Probable mechanisms of the magnetic field influence on gene expression and virus-host interactions.

We predict several possible ways in which magnetic fields can modify gene expression, alter the NF-κB signaling and thus, influence virus-host interactions (Figure 11). First, it should be noted that genes encoding proteins of Rel/NF-κB/IκB pathway belong to an extensive set of clock-controlled genes [101]. As such, they are exposed to transcriptional regulation by the CLOCK/BMAL1 complex, whose activity in turn depends on binding with CRY, and correspondingly the magnetic field-dependent function of Cryptochrome (see Figure 6).

Interestingly, the transcription complex CLOCK/BMAL1 also regulates the transcriptional activity of the glucocorticoid receptor [102]. Furthermore, it is known that the relative abundance of CBP in the nucleus can influence NF-kB and glucocorticoid receptor transcriptional antagonism [103]. At the same time, CBP binds BMAL1 very closely to the CRY binding cite. Thus, we can hypothesize that magnetic fields can influence the duration of the CBP-BMAL1 interaction *via* regulation of the functional activity of CRY. In turn, prolonged CBP retention by the CLOCK/BMAL1 transcription complex may influence the equilibrium between pro-inflammatory and immune-suppressive signals in cells and tissues. Together, the data suggest that the major circadian complex indirectly influences the functions of every organ and tissue inside the body through modulation of both: the NF-κB- and glucocorticoids-dependent signaling pathways.

Another way magnetic field regulation of the NF-κB target genes provides participation of the magnetic field-sensitive CRY in mechanism of active transcriptional repression is implemented by CLOCK/BMAL1/CRY complex [60] (see chapter 4.2). For example, induction of NF-κB target genes in response to stress can occur only under those conditions (time of the day and magnetic field parameters) when CLOCK/BMAL1 is functioning as an activator and does not block the activity of other transcription factors. And *vice versa*, when the CLOCK/BMAL1/CRY complex acts as an active transcriptional repressor, the induction of NF-κB target genes and other stress-induced genes will be attenuated or completely suppressed. Therefore, the pattern of stress-induced gene expression and an organism’s response to stress will depend on the functional activity of CRY, which in turn can be regulated by magnetic fields. This mechanism allows external magnetic fields to produce global regulatory effects on gene expression. Indeed, the ability of electromagnetic fields to affect non-circadian gene regulation has been confirmed in numerous reports [18,28,60,104–106], reviewed in [29,77]. Definite immunological alterations caused by EMF exposure have been described (reviewed in [107]). A significant decrease in leukocyte, erythrocyte, lymphocyte and monocyte counts in mice chronically exposed to weak EMF was detected [108]. The intensity of EMF in these experiments was 5.0 microT, several times weaker than the geomagnetic field strength. In human populations, chronic exposure to weak ELF EMF (50 Hz, 0.2–6 microT) resulted in a significant fall in total lymphocytes and the T-helper subpopulation, whereas there was an increase in NK cells. Among other manifestations of the EMF exposure, there was an increase in the degree of neurovegetative disorders (*i.e*., physical fatigue, psychical asthenia, depressive tendency, irritability) [109]. Significant elevation of phagocytic activity, stimulation of free radical release and IL-1b production were detected in macrophages exposed to an ELF magnetic field [110].

We believe that the data we have presented concerning the probable influence of specific external magnetic fields on immunological processes and virus replication *via* the NF-κB and other signaling pathways allows us to put forward a possible role of geomagnetic field fluctuations, and correspondingly, solar activity cycles as factors capable of influencing the occurrence of influenza (and possibly other) epidemics. It is worth mentioning that several viruses, including HIV, have binding sites for NF-κB that control the expression of viral genes, which in turn contribute to viral replication and/or pathogenicity. In the case of HIV-1, activation of NF-κB may, at least in part, be involved in activation of the virus from a latent, inactive state [111]. This indicates the theoretical possibility of magnetic field effects on the processes of HIV activation and replication.

Broadly speaking, NF-κB-controlled pathways are relevant to many human diseases. NF-κB is chronically overexpressed in inflammatory diseases (arthritis, sepsis, asthma, among others). There is evidence for a critical role of NF-κB in carcinogenesis, and NF-κB is a key mediator of chemotherapy resistance, as well as having a major role in tumor development, particularly in its early phases [112]. Therefore, blocking NF-κB can inhibit tumor cell proliferation, increase their sensitivity to the action of anti-tumor agents or induce apoptosis. This emphasizes the importance of NF-κB inhibitors as immunotherapeutic agents for chronic inflammation and suggests that these reagents might prevent, or at least inhibit, chronic inflammation-associated tumorogenesis [113]. Therefore, the possibility of using external magnetic fields for the regulation of Rel/NF-κB/IκB expression by involving the magneto-sensitiveness of Cryptochromes looks promising.

The transcription regulatory effects of magnetic fields can be manifested in many different ways. Correspondingly, their application in both prophylactic and therapeutic ways also calls for extensive research in this field. Success in this direction is already evident. For example, ELF EMF treatment has been used to stimulate endothelial cells proliferation, and in the activation of wound healing and angiogenesis. In these experiments, the stimulatory influence of ELF EMF on VEGF receptor 2 expression was demonstrated [105]. Exposure to ELF magnetic fields triggers the expression of cardiac lineage-promoting genes GATA-4 and Nkx-2.5 and enhanced prodynorphin gene expression in mouse embryonic stem cells [18]. These effects play a key role in cardiogenesis at the transcriptional level and cause a remarkable increase in the yield of embryonic stem cell-derived cardiomyocytes.

## 8. Conclusions

We believe that the data presented here allows us to conclude that most living creatures and many types of cells can be sensitive to weak magnetic fields due to magnetosensitive proteins—Cryptochromes, which are important regulators of the major circadian transcriptional complex CLOCK/BMAL1 activity. The transcriptional repression function of CRY is apparently highly responsive to weak MF because of radical pairs, which periodically arise in the functionally active site of CRY and mediate the radical pair mechanism of magnetoreception. Thus, environmental exposure to EMFs can interact with fundamental biological processes in the body, confirming the possible bio-informational and bio-regulatory functions of electromagnetic radiation.

The presented data also indicates the potential use of magnetic fields in modifying gene expression patterns and programs in different cell types, including stem cells, without the aid of gene transfer technologies. Thus, the proposed mechanism of the magnetic field’s bio-regulatory effects seems to offer new options and molecular targets for development of new biotechnological, therapeutic and prophylactic strategies, including those of epidemiological significance.

Viruses are indispensable part of the evolution process machinery. They act both: as a factor of genomic diversity increase and as a natural selection factor. Taking into account a probable entrainment role of Solar activity fluctuations in regulation of genome expression and influenza epidemic cycles, we can suggest regulatory/entrainment role of the Solar cycles for Earth life (biosphere) microevolution.

According to the Space Weather Prediction Center NOAA/SWPC, the next solar activity maximum is expected in the year 2013 [114]. Following the main idea of this review, it is suggested that from 2011 until 2015 solar regulatory influences and concomitant geomagnetic field fluctuations may predispose to genetic and immunological alterations favorable to influenza epidemic spread.

Dynamics of our knowledge in the field reviewed in this paper confirms and supplements ideas of Chizhevsky [1] about the electromagnetic nature of communications between spatially distant components of the single whole (integrated) system comprising our Biosphere and the Sun.

Based on this foregoing analysis of the pertinent scientific knowledge and our concept, it is possible to formulate the following principles in a new scientific area of research as “Physical Epidemiology”:

1. Perturbations of cosmic plasma flow (such called star or solar “wind”) caused by Solar activity alterations induce periodic changes in the geomagnetic field that is among immediate regulatory signals for Solar-correlated cycles in Biosphere, including cyclic modulation in gene expression patterns of living beings. This solar activity-dependent regulation of gene expression can clearly lead to immunological, epidemiological and other consequences.
2. Among the universal biological “antennae” of the magnetic regulatory signals, it is important to include proteins of the Cryptochrome family and Ca^2+^ signaling pathways. Cryptochromes can function as “epigenetic sensors” of the geomagnetic field fluctuations, the magnetic field-sensitive part of the epigenetic regulatory mechanism.
3. The radical pair mechanism of magnetoreception (effects related to spin chemistry) can account for high magnetic responsiveness of Cryptochromes, which incorporates the radical pairs in their functionally active sites.
4. CRY are transcriptional repressors of the major circadian complex CLOCK/BMAL1, therefore magnetic fields *via* modulation of CRY function can influence circadian gene expression, modify activity of NF-κB- and glucocorticoids-dependent signaling pathways.
5. The pattern of stress-induced gene expression and organismal response to stress will vary depending on the functional activity of Cryptochromes, which in turn may be regulated by magnetic fields and, correspondingly—by solar activity cycles.
6. We hypothesize that solar cycles are able to both regulate, entrain processes of biological microevolution and to tune biological rhythms (bio-clocks) in living beings implementing mechanisms stated above.

## Abbreviations:

EM: electromagnetic
EMF—: electromagnetic fields
ELF EMF—: extremely low frequency EMF
CRY—: Cryptochromes
CBP—: CREB binding protein
